# Positionality and intersectionality in environmental debates: duoethnography as a method to know (from) the margins

**DOI:** 10.1080/25729861.2025.2484118

**Published:** 2025-06-18

**Authors:** Jose A. Cañada, Joaquín Valdivielso

**Affiliations:** a Centre for the Social Study of Microbes, Faculty of Social Sciences, University of Helsinki, Helsinki, Finland; b Department of Philosophy and Social Work, University of the Balearic Islands, Palma, Spain

**Keywords:** Duoethnography, environmentality, intersectionality, knowledge, tourism, Duoetnografia, ambientalidade, interseccionalidade, conhecimento, turismo, Duoetnografía, ambientalidad, interseccionalidad, conocimiento, turismo

## Abstract

The Balearic Islands (Spain) are one of the main tourist destinations in the world. Since the 1950s, the tourism industry has had a considerable impact on the environment, resulting in a strong tradition of environmental activism. Balearic environmental movements have engaged in the production of knowledge and technical evidence to sway debates for legislation in favor of protecting the environment. We argue that the limited involvement of migrant populations in such movements reflects the class and ethnolinguistic barriers that inform the creation of human–environment attachments. Our work reveals two methodological precarities: the lack of access in migrant populations to know the environment and our own capacity to track such histories. To tackle them, we rely on duoethnography as a method, using our own intergenerational experiences as descendants of migrants as empirical material. With them, we reflect on how our participation in public debates is mediated by our positionality as determined by the intersection of class, ethnic, and linguistic identities. Using the notion of environmentality, we illustrate the onto-epistemic openings and limits linked to identity that historically characterize environmental struggles, in turn showing the potential of duoethnography to contribute to scholarly debates in contexts of methodological precarity.

## Resumo

As Ilhas Baleares (Espanha) são um dos principais destinos turísticos do mundo. Desde a década de 1950, a indústria do turismo teve um impacto considerável no meio ambiente, algo que motivou uma forte tradição de ativismo ambiental. Movimentos ambientais das Baleares estão envolvidos na produção de conhecimento e evidências técnicas para influenciar a legislação em favor da proteção ambiental. Aqui argumentamos que o baixo envolvimento das populações migrantes em tais movimentos reflete as barreiras de classe e etnolinguísticas que informam a criação de vínculos humano-ambientais. Nosso trabalho revela duas deficiências metodológicas: a falta de acesso das populações migrantes ao conhecimento do meio ambiente e nossa própria capacidade de rastrear essas histórias. Para abordálos, usamos a duoetnografia como método, usando nossas próprias experiências intergeracionais como descendentes de migrantes como material empírico. Por meio dessas histórias, refletimos sobre como nossa participação em debates públicos é mediada por nossa posicionalidade, ela própria mediada pela interseção de identidades de classe, étnicas e linguísticas. Com base na noção de “ambientalidade,” ilustramos as aberturas e os limites onto-epistêmicos ligados à identidade que caracteriza historicamente as lutas ambientais nas Ilhas Baleares, mostrando, por sua vez, o potencial da duoetnografia para contribuir com os debates acadêmicos em contextos de precariedade metodológica.

## Resumen

Las Islas Baleares (España) son uno de los principales destinos turísticos del mundo. Desde la década de 1950, la industria turística ha tenido un impacto considerable en el medio ambiente, algo que ha motivado una fuerte tradición de activismo ambiental. Los movimientos ambientalistas baleares se han involucrado en la producción de conocimiento y evidencia técnica para influenciar la legislación en favor de la protección ambiental. Aquí argumentamos que la escasa implicación de las poblaciones migrantes en dichos movimientos refleja barreras de clase y etnolingüísticas que informan la creación de vínculos humano-ambientales. Nuestro trabajo revela dos precariedades metodológicas: la falta de acceso de las poblaciones migrantes a conocer el entorno y nuestra propia capacidad para rastrear dichas historias. Para abordarlas, usamos la duoetnografía como método, utilizando como material empírico nuestras propias experiencias intergeneracionales como descendientes de migrantes. Mediante estas historias reflexionamos sobre cómo nuestra participación en debates públicos está mediada por nuestra posicionalidad, en sí misma mediada por la intersección de identidades de clase, étnicas y lingüísticas. A partir de la noción de “ambientalidad,” ilustramos las aperturas y límites onto-epistémicos ligados a la identidad que históricamente caracterizan las luchas ambientales en Baleares, mostrando a su vez el potencial de la duoetnografía para contribuir a los debates académicos en contextos de precariedad metodológica.

## 1 Introduction

The Balearic Islands are one of the main tourist destinations in the world. Since the 1950s, the construction of tourism infrastructure, the abuse of island resources, and urban sprawl have spurred controversies due to their environmental impact. Scientific, public, and private actors contribute to those controversies by producing knowledge and technical evidence to support or hinder initiatives for environmental regulation. Unsurprisingly, the region has a strong tradition of environmental activism that has pushed much of the environmental research done in the islands. Environmental movements have helped to put conservation at the center of the public agenda through “knowledge transfer” and the “transformation of mentalities” (Serra and Nigorra 2023, 15), including the formation of citizen epistemic communities linked to recent mobilizations (Valdivielso and Moranta 2019). Conservation debates have been mainly configured around the defence of the *territori –* a term that discursively links land, territory, landscape, and environment (Valdivielso 2010) – and a stark opposition between that which is “ours ” – island, Catalan-speaking ethnolinguistic identity, traditions, values – and “theirs ” – extractivist interests in the economic grabbing of Balearic landscapes and resources – placing identity in a central role in Balearic politics.

Contributing to environmental debates is a key way of public participation in the Balearics and doing so requires not only being well-versed in the terms of the debate, but also having an attachment toward the environment that motivates caring for it (Cañada 2024), an attachment deeply interlinked with the social, regional, linguistic, and cultural identity. The background to such attachments is complex: structural changes in the Balearics include a massive demographic change. Around half of the resident population was not born on the islands and at least 21% is foreigner. A whole range of terms distinguish between different forms of belonging according to different ethnolinguistic, racial, class, and national cleavages but, on the whole, the demonym of each island – *mallorquí, menorquí, eivissenc, formenterenc –* normally designates just the descendants of those who were resident in 1950 – no more than a third of the population (Jordà, Colom, and Mayol 2016).

This configuration results from the Balearics’ status as one of the biggest destinations for unqualified migrant workforce in Spain. From the mainland in the early days of tourism, and more recently from the Global South, migration has diversified the archipelago’s identity landscapes and has played an indispensable role in its economic development. The first waves, between the 1950s and the 1990s, came from impoverished rural areas of the mainland and led to the establishment of segregated neighborhoods (*barrios*)^1^, most of them in the rapidly growing capital city of Palma (González and Somoza 2004). These *barrios* marked a difference in economic class while hindering the integration of migrants into a society with a strongly differentiated linguistic, cultural, and historical identity. Such differentiation is evident in the concept of *foraster*, Catalan word to pejoratively describe Spanish-speaking migrants from the mainland. Unlike other comparable cases such as Catalonia, where already in 1964 the social-journalistic study *Els Altres Catalans* (Candel 1964) reaffirmed the hybridization and the figure of the migrant *xarnego*, Spanish speakers have not participated in the construction of the mainstream insular identity (Jordà, Colom, and Mayol 2016). With Balearic politics gravitating around notions of environmental impact and territorial identity, the limited integration of migrant groups is reflected in the demographic composition of environmentalist grassroots movements, which have been predominantly Catalan-speaking and middle-class, identifying with the territory’s “own” tradition, and rural imaginary (Mayol 2021; Picornell 2023). Activist groups are key because they provide a voice to citizens to participate in technoscientific debates about environmental matters, also serving as an educational resource and a socialization tool to get to know and care about the more-than-human relations that sustain the island.

This lack of diversity in the development of environmental identities connects with two literature gaps that motivate this article. On the one hand, research on environmental movements and conflicts in the Balearic Islands does not address the relationship between activism and the socio-demographic profile of the actors involved (Ferrer 2015; Janer 2022; Serra and Nigorra 2023). This deficit reflects an unproblematized presupposition of the social construction of environmental conflicts: that they are led by native populations with roots in the land who mobilize to defend it (Mayol 2021). The exception is provided by some works on Ibiza, which recognize the role of well-off residents of European origin in citizen mobilizations in environmental conflicts (Miró 2020), and a recent study about the underrepresentation of lower classes in social movements (Amrhein and Langer 2025). Secondly, a review of recent literature on identity in the Balearics – scarce until a decade ago – reveals a need to overcome the essentialist ethnolinguistic model and to recognize the new hybrid subjects that destabilize it. However, these studies tend to assume an “own culture,” framing the process of cultural interaction with otherness in terms of assimilation rather than hybridization (Jordà, Colom, and Mayol 2016; Vives and Vicens 2021). Probably the most successful attempt to give voice to new forms of hybrid agency is Mercè Picornell’s (2023) analysis of self-image in different cultural productions. Nevertheless, these studies tend not to refer to class or migration, only represented vicariously and at the margins.

As descendants of working-class migrant families, these gaps in literature promptly resonated with our experiences of access to knowledge production and environmental participation. We both are academics working on topics related to environmental research in the social sciences and humanities and Joaquín has a long history of activism in the Balearics originating in 1996. Through our different projects, we have participated in the debates and knowledge production mentioned above. Realizing the absence of migrant perspectives in Balearic academia we understood that filling that gap required significant original research, a project we felt unable to tackle due to a mix of academic precarity, lack of time, and resources. Consequently, we turned to our own experiences as sources of information to illustrate those dynamics of identity formation and environmental participation while also helping to understand the way migrant groups growing up in the margins manage to participate in knowledge production about environmental matters. Doing so, however, required methodological inventiveness in response to the mentioned limitations and the consequent methodological precarity.

This article aims at exploring the methodological advantages and limitations of using the researcher’s own stories as data, contributing to illustrating challenges in knowledge production and participation from the margins. In Section 2, we identify duoethnography as the most suited methodological approach for this and discuss its potential to study the social, material, and political precarities of knowledge production. Section 3 presents the central knots and narratives featured in our duoethnographic conversations about our histories of environmental engagement and knowledge production, and how they are connected to our specific positionalities and intersectional experiences as descendants of working-class migrant families. Section 4 presents a theoretical discussion of those narratives in order to illustrate the capability of duoethnography to produce findings that can be integrated into existing scholarly debates. Thus, while the article focuses on a methodological contribution, the theoretical discussion of our empirical material helps to prove the viability of duoethnography as a precarious method. By means of that discussion we engage with international debates about environmentality and knowledge production in Science & Technology Studies (STS), taking advantage of the Balearics’ status as a laboratory for observing the impacts of tourism capitalism (Valdivielso and Moranta 2019). We conclude by vindicating duoethnography’s capacity to produce academic knowledge. Besides the benefit of making possible the conducting of research in low-resource settings, it can visualize marginal positions in academic debates while forcing researchers to rigorously reflect on their positioning as knowledge producers, both from a perspective of marginalization and privilege.

## 2 Material and methods: duoethnography

As mentioned in the previous section, given the lack of resources to kickstart a project of the necessary scale to address the formulated gap through more conventional methods, we commenced a search for more accessible alternatives. Our search took us to “duoethnography” (Sawyer and Norris 2013), a collaborative research technique whereby two (or more) researchers juxtapose their life histories to understand the multiplicity of a given phenomenon. In our case, we used our intergenerational experiences as descendants of migrants from the Spanish mainland to the island of Mallorca^2^ in the 1960s. Choosing duoethnography is a way to deal with a double methodological precarity. The first one has to do with the barriers that migrant neighborhoods faced in accessing and knowing the most valued natural environments. The segregation characteristic of migrant areas is here present both spatially and epistemically since knowledge about the island is key to the way education and socialization into environmental matters articulate discourses of belonging and identity imaginaries. Knowing without belonging requires precarious approaches, as we illustrate in the next section. The second precarity has to do with the underrepresentation of migrant perspectives in Balearic scholarship. In researching such gaps, we realized our limited capacity to kickstart a new line of research considering the precarity that characterizes contemporary academia (Burton and Bowman 2022). Given that our own histories are fitting study cases, we decided that putting our own family and personal histories at the center is a way to ensure this discussion does not fade away despite the limited interest in Balearic perspectives situated in the connection between hybrid identities and specific modes of environmental participation.

Duoethnography allows us to explore in great detail the different moments in which those hybrid identities crystallize into participation and knowledge production, a level of detail and knowledge of the data that contrasts with the unavoidable gaps present in more traditional methods like surveys and personal interviews. We are therefore able to draw on long histories of engagement with the topic at hand. Such engagement is not a promise of enhanced contact with reality but rather a chance to participate in re-doing it (Puig de la Bellacasa 2009). With this method, we let our own life histories become sites of inquiry. Through dialogic narrative, we reflect on our experiences of knowledge-seeking and public participation as mediated by the intersection of our own class, ethnic, and linguistic identities as descendants of working-class migrants. Part of that reflection involves noticing that ours are histories of upward social mobility where we both, through education and parental labor, have accessed positions of academic expertise and are able to research and study environmental matters from critical perspectives. Still, we aim at problematizing what expertise comes to count and how knowledge produced from the *barrio* can also contribute to a different understanding of what protecting the *territori* might mean.

Duoethnography also entailed challenges that we kept in mind from the beginning. The first, deeply linked to our interest in intersectionality, was to acknowledge that our own perspectives could be both illustrative and limited by our own positionality, something we consider in drawing our conclusions. We both were raised as white non-disabled boys (even if Jose has since come out as nonbinary) so, although we did witness (and discussed during our duoethnographic practice) barriers in accessing nature in our *barrios* associated to classic intersectional categories such as race, gender, or disability (Collins 2019), we acknowledge that our own experiences are not fully comprehensive. Indeed, some other groups suffer most from structural inequalities, such as racialized informal workers, facing enormous barriers to having a public voice and participating as full citizens in environmental debates (Amrhein and Langer 2025). This is a key limitation of the selected methodology but also a key aspect of the precarity associated with this work that allows for constructive conversation with the rest of the thematic collection: addressing our questions with enough time and human resources would allow to include other voices and reflections that would add nuance to the way migrant, ethnolinguistic, and class identities converge with other intersectional categorizations. Certainly, one of the unavoidable conclusions of this work is the need for complementary studies able to attend to the breadth of categorizations and experiences that help understand the diversity of histories and engagements with the environment in the Balearics.

A second challenge is the “making” of our histories. Doing duoethnography has both practical and performative aspects, which entail two risks. First, gathering our memories, reflecting them on each other, and building coherent narratives around them involves a risk of engaging in modes of storytelling that encourage “hero or victim sagas” with “self-congratulatory tone” (Breault, Hackler, and Bradley 2012, 122). While not entirely avoidable, we minimized the risk by establishing beforehand the topics to be discussed, including intersectional categories such as disability, gender, or race that would force us to discuss both our disadvantages and privileges. Furthermore, when one of us was telling their story, the other was assigned with critically exploring and challenging the topics as they came up in a kind of two-way interview that followed the tenets established by Sawyer and Norris (2013). These tenets as well as the specific technical steps we followed to register and analyze our histories can be found in “Annex 1.” The results of the duoethnography are therefore interactively performative: together we situate our own histories but also produce an account of a sociomaterial reality in which those histories take place. Rather than inferring what were the knowledge opportunities for similarly positioned others, we prioritize our “experienced access and doing” as a way to highlight the intrarrelation that characterizes the connection between people and territory (de la Cadena, Risør, and Feldman 2018).

Duoethnography tends to be presented as reconstructed conversations foregrounding its dialogical dimension (Norris and Sawyer 2012). However, the space constraints of a journal article force us to present our material in a different format. Instead, we present a shared narrative, including common experiences and points of divergence. This involves a risk of mixing voices, something we minimize by using the pronoun *we* when referring to common experiences/positionalities, the pronoun *they* for those concerning only Jose, and *he* for those concerning Joaquín. The topical breadth of our discussions is also limited by space. Our conversations were lengthy and went into great detail of how our experience of engagement with nature and the will to participate in knowledge production might have connected with situations or episodes the relevance of which was not evident right away. To keep the presentation of our results concise and relevant for the context of this article, we focus on five themes summarized in the next section. Conversational context for the brief excerpts used to illustrate key points can be found in “Annex 2.”

## 3 Results

### 3.1 Migration and childhood

Our histories are inescapably linked to the migration histories of our families. Joaquín arrived in Mallorca as a baby when his family moved from Bilbao in the early 1970s. His father worked as an elevator mechanic, and, in an island undergoing an economic boom thanks to tourism, this was a sought-after professional profile. However, the sector’s subsistence salaries led Joaquín’s family to move to the recently established migrant *barrios* spanning around the city of Palma:

[Son Cladera] was a newly built *barrio*. At the time, in the end of the sixties, seventies, Son Roca, Son Gotleu, Son Oliva were built, in other words, lots of *barrios* that were built and that became ghettos for the migrant population. They were not closed ghettos, in the sense of the racially segregated neighbourhoods of the United States, but there were invisible barriers that created a very different culture, very different social networks between the migrant *barrios*, one hundred percent of which were mainlanders, I would say, and the other *barrios* of the population already living in Mallorca for generations. (Joaquín; E1)

This type of neighborhood established a clear integration barrier. They were

areas of socialization with very particular features, where, for instance, the use of Spanish predominates, Catalan or Majorcan is hardly ever heard, and inhabited by poor, working class populations. Particularly in the Polígono de Levante, with a high level of marginalization and even crime. (Joaquín; E1)

Jose’s family would move to a similar *barrio* in Palma’s west side in the beginning of the 1980s. Jose’s parents, both from Granada, had arrived in Mallorca as teenagers in the previous decades, escaping the poverty and repression of the Andalusian countryside. They were hired as unskilled labor in the booming hotel industry.

### 3.2 Barrio *and urban nature*

These migrant backgrounds are central in a territory where identity is such a fundamental element in how society interacts with its own surroundings. We both reflected on how we felt not only unable to develop a Majorcan identity, but we were also denied the possibility of developing the identity of our parents as their migration marked a disconnection with their cultures of origin: we were not Majorcans, and even less *granaínos* or *bilbaínos*. So, what territory should we care about? This made us look at the natural environments that surrounded us as kids and teenagers, which was a very similar experience for both of us. Our *barrios*, located in the outskirts of Palma, were surrounded by non-urban areas but we both remember little to care for there:

the natural spaces we had access to were spaces like […] the wasteland behind the Majorcan part […] of Son Rapinya, there was a wasteland which [went all the way] to Son Vida and to the Genova military base, and all that was a wasteland and a dump, there were a lot of electrical appliances lying around, fridges, washing machines, and so on, there were also syringes, etc. So, well, we were forbidden to go there when we were kids. Then there were also wastelands towards La Vileta, towards Son Vida …, there was a lot of wasteland, a lot of wasteland, but it was all very poorly maintained. (Jose; E2)

These “degraded environments,” as Joaquín (E2) described them, contrast with access in Catalan-speaking, middle-class communities who, through their inhabiting of rural and coastal areas and thanks to activities organized by traditional cultural and environmental grassroots movements, had regular access to what in Mallorca can be described as “natural spaces of high environmental value.” Our experiences of nature were, as these excerpts illustrate, of degradation, violence, danger, and absence of care toward people, animals, and the environment. Not only were those spaces not cared for, but also served as the margins of society since they were usually occupied by at-risk groups, such as drug-addicts:

Without having the language to express it, you grow up with the feeling that you are in a degraded environment. The contact with nature that we had in our neighbourhood was, in the Polígono de Levante in particular, the wasteland in front of our house, where there was unused machinery, where it was very easy to have an accident, where there were syringes, or, walking a little, the beach of Can Pere Antoni, where all the junkies of Palma went to *shoot up*. (Joaquín; E2)

### 3.3 Classed nature

This contrast in access between rooted “Majorcan” and *foraster* migrant populations and the resulting opportunities to develop an affective relation with nature is what we referred to as “classed nature” in rethinking our experiences, something that went into our teenage years and young adulthood. As we grew up and gained independence and mobility, we started discovering the Mallorca outside the limits of our *barrios*. While, for our families, summer was a time for working, dictated by the seasonality of the island’s tourism industry, for others it was a chance to enjoy the island’s weather and natural spaces through leisure activities:

Well, what did you do in the summer? Work, I mean, since we were children we worked in the summer, either tutoring, or when I was fifteen, my father took me to a building site […] where I spent the […] summer helping him. And so did my siblings. And, of course, our middle-class or Majorcan peers, let’s say, *estiuejaven*, they would go to a summer apartment or to the house in the village, […] usually with their grandparents, enjoying the summer, swimming in the cistern or at the beach, and obviously without having to work. And it was something that shocked me, I didn’t understand. (Joaquín; E3)

This shows how intersectionality is key in order to understand dynamics of access to nature: it was not in isolation that our lack of *mallorquinitat* (“Majorcan-ness”) – with its language, culture, and knowledge of the island –, or our *barrio* working-class background, limited our access to nature, but a combination of those factors that turned the island’s seasonal dynamics into another inequality marker. Once we were both of working age, being able to study was dependent on savings during the summer season. Both of us paid for our studies through summer work. This contrasts with the experiences of the upper classes where *estiuejar* (summering) is a common practice. The fact that in our conversations, we instinctively used the Catalan word *estiuejar* offers an idea of how language intersects with class here: *estiuejar* in Mallorca is a class practice that intersects with culture and language. This has a direct impact on the development of an environmental attachment since *estiuejar* offers direct contact with non-urban environments and the possibility of developing an affective and emotional attachment toward them.

### 3.4 Academic education

In repairing that relationship with nature, limited almost exclusively to degraded urban spaces, education was a defining factor. Especially higher education served to meet people from different cultural, linguistic, and class backgrounds. However, here we noticed a generational difference in the way educational institutions offered access to natural spaces. Jose’s first experiences of non-urban nature were tied to day trips in primary school:

Jose: I don’t know if there’s going to be a […] generational contrast in terms of [how and] when you first step out of the *barrio*. In my case it was when we did school trips. I don’t know if it was the same when you went to school […]. Did you make day trips with the school?

Joaquín: We did some in high school, but not in primary school. I don’t remember [doing any] as a child.

Jose: OK, so for example, there you have an important generational difference […]. Since we were little, from primary school, we went to the Esporles Farm, we went to the Lluc monastery, and you did a bit of walking, and they explained things about the Tramuntana mountains. That had to be mediated by an institution, which is the school, so that I could have access to it. (Jose & Joaquín; E4)

Joaquín’s experience, on the other hand, is tied to a later stage in life. While he remembers some day trips to visit family friends in the then still virgin northern part of the island, these are exceptional and vague memories. For Joaquín spending time in natural spaces is linked to the access to a different social world like the one offered by university life.

When I socialize in the university circles, I find myself in an environment [that is], let’s say, very alternative, where I experienced immersion in a Catalan-speaking environment […] and this opened up a new social world for me. As part of this there was also a greater knowledge of Mallorca, in its different aspects, socially, culturally, and particularly in terms of the landscape and environment. We began to go on excursions, I began to see places I had never seen before, and this increased a perception that I had had since I was very young of becoming aware of the poverty, from a sensory point of view, of life in an urban *barrio*. (Joaquín; E5)

Knowing the island for Joaquín requires changing his own positionality: from a migrant working class, Spanish-speaking neighborhood to a middle-upper class, Majorcan and Catalan-speaking academic environment, opening life beyond the *barrio*. Jose’s narrative is not entirely dissimilar, although it involves leaving the island to study in Barcelona. Once there, Jose explains that self-presenting as Majorcan outside the island but not fitting into what mainlanders thought Majorcans are and sound like makes them rethink their own identity: what does it mean to be Majorcan? What do Majorcans speak and how do they speak?

I went to university in Barcelona, Barcelona is also a big city, where I still very much had a spatial identity linked to the urban. I loved the idea of living in a bigger city. [But] I also started to become more aware of the island as an island, and not just Palma as a city. I began, just because I had to present myself as a Majorcan, to think of my identity […] in relation to the whole island, and not just the *barrio*. (Jose; E6)

This developed place awareness pushed Jose to want to know the island and what it contained. For Jose, studying in Barcelona is a challenge to their Majorcan-ness, and makes them question their attachment to the island. This motivates them to claim a cultural and territorial connection with the island, get to know it and explore it. This development of a renewed Majorcan identity is reaffirmed when Jose moves to Finland to do a PhD and eventually becomes integrated socially, culturally, and linguistically in a way that contrasted with the lack of integration that characterized their childhood and youth in Mallorca. This produces a further motivation to claim a connection to the island and brought up questions: why is Jose more able to claim a Finnish identity? Why do they feel more confident speaking Finnish than Catalan, their second language? For Joaquín, it was a move in the opposing direction. His “Majorcan” identity was reaffirmed by his integration into “Majorcan” society: first as a student in the local university, then as a doctoral candidate, until reaching his current professorial status. Going to university in the Balearic Islands gave him the opportunity to socialize with the better-off, Catalan-speaking class that has a clearer identity link with Majorcan natural spaces.

### 3.5 Class betrayal

Our last category has to do with how the resignification and problematization of our own roots are only possible through a redefinition of our own social positionalities: Joaquín’s attendance to university makes it possible for him to not only experience natural spaces in “Majorcan” terms, but also integrate himself in grassroots environmentalist movements, those that we described in the introduction and that are predominantly populated by, again, better-off, Catalan-speaking communities with Majorcan roots. Later, during the last decade, he found himself representing those movements in the public sphere, even though he did not embody the typical traits presupposed in such milieu.

This “escape” from the *barrio* concerns both space and identity: both of us gather the necessary resources to perform a *mallorquinitat* that is much closer to the cultural and linguistic *mallorquinitat* to which we did not have access during our childhood. This makes us start building a different relationship with the spaces of high environmental value on the island that leads us to want to get involved in their conservation. This is, in the sense formulated by Marisol de la Cadena (2014), an “onto-epistemic opening.” The sociomaterial processes that lead to a lack of access to natural environments are onto-epistemic limitations that constrain our ability to engage with them in caring ways or, in the best case, delay it. The limits also work in the other direction: urban nature is disregarded and expelled from the territory to protect because of its non-pristine, “low value” condition. This move divides the environment into two – high and low value – forcing different types of people-territory intrarrelations (de la Cadena, Risør, and Feldman 2018) among different communities. Approaching those spaces also involves a distancing from the marginal, working class, migrant *barrios*. Accessing academic titles and better-paying jobs often entails giving up on performative class markers like accents, body aesthetics, or fashion (Vasallo 2021), creating a tension between the mandate for social mobility and the will to not renounce one’s class roots.

[This] also creates a bit of a tension in me. I mean, it’s like abandoning the *barrio* […] or like not paying attention to the problems I grew up with […]. I have now started to pay more and more attention to [Majorcan natural] spaces because of the awareness that living in a much more rural country [like Finland], with a very rich natural environment, very well preserved, has given me […]. It makes me want to return to Mallorca, not just for holidays, when I come to see my family, but with a project that is a little more aligned with my intellectual concerns. […] I had never thought of it in those terms, […] that sensorial poverty that you were talking about in your *barrio*, […] for me it explodes when I go to Finland and I see landscapes, lakes, incredible forests […]. So, it forces me to rethink the weight that I have given to the urban, to the big city, to the capitals […] (Jose; E7)

And so that escape from the neighborhood also means a kind of return in both cases. Sennett and Cobb (1972) spoke of the hidden injuries of the working class. Many of the insecurities and barriers we have presented in these results can be read as examples of open wounds: insecurities linked to our identity and shaped by experiences of discrimination and lack of access. Our reflection emerges from a desire to overcome and, continuing with the metaphor of the open wound, from a desire to heal. Such healing, for us, takes the shape of understanding the kind of travel necessary to start producing knowledge from the margins while acknowledging the inadvertent incorporation of a kind of class betrayal into our estrangement.

## 4 Discussion

Earlier, we set to explore the advantages and limitations of using our own histories as research data as a way to deal with methodological precarity, an objective that is methodological in essence. However, we believe achieving it entails also testing duoethnography’s ability to contribute to scholarly debates through rigorous discussion with other literatures. Given the topic of the collection and our formulation of the Balearic setting in the introduction, we choose to engage in conversations about access to environmental knowledge production from the margins. The way such margins are epistemically and spatially defined in our narratives is central to such discussions. In our narratives, margins are defined in relation to a center of environmental research and green activism with Majorcan roots. However, it is important to acknowledge that such center is a margin in itself. From the point of view of the hegemonic vision of a tourist society, activism and environmental research have been placed in a position of subalternity. There, caring for the environment means challenging the common sense of infinite growth and extractivist capitalism. Yet, the emergence of environmentalist subjectivities in such a society implies a matrix of normalization that privileges some specific places of enunciation, while others, such as that of our *barrios*, remain silent. To describe such contexts, Dumoulin, Kleiche-Dray, and Quet (2018) use Agrawal’s (2005) notion of “environmentality” to explain the production of subjectivities through environmental governance that gives rise to a classification of populations according to the value of their ecological practices. Following them, Latin American Anti-colonial, Post-colonial, Subaltern and Decolonial research programs allow to theoretically formulate these dialectics of voice selection to trigger a dialogue between different knowledges to deconstruct colonial epistemologies and re-write environmental discourses from below, including the perspective of the “environmentalism of the poor,” in the famous formula of Joan Martínez Alier (2002), as a defence of the local livelihoods against the market commodification of their ecosystems. However, this explanatory model is too simple for cases such as ours.

In our case, the *barrio* emerges as the place where that environmentalism of the poor can take place. However, the *barrio* as urban environment also lacks access to the background environmental conditions necessary to participate of conflicts around dispossession and the loss of livelihoods. Moreover, the position privileged by environmentality self-identifies as native against the migrant *foraster* from the *barrio*, sometimes lumped with the ethnolinguistic otherness of affluent *estrangers*, critically disregarding class. Such environmentality is not the sort of technocratic institutional governance that the concept itself tends to assume (Luke 2012), but still works as a normalizing matrix, ranking voices according to their rootedness in the *territori*. Our biographical experiences make explicit an emerging subject silenced and dislocated from the “native” community’s privileged environmental voice – as introduced in the beginning of the article – that emerges around the idea of saving the islands by protecting or defending a *territori* (Mayol 2021) consumed by urbanization – to house *migrant* workforce *–* and touristification – or capitalist extractivism led by higher classes and affluent newcomers. In this variety of environmentality, *territori* operates as an empty signifier, which can simultaneously refer to the defence of a landscape, of a pre-tourist ethnolinguistic community substituted by migration, and of the rural versus the urban (Valdivielso 2010). In this context, the dominant environmentality has been shaped by the images of low anthropized emblematic landscapes, and of a folk *mallorquinitat* that opposes the homogeneous autochthonous “us” rooted in the land to the colonizing other. Both dimensions are captured in the omnipresent image of the “lost paradise” (Ferrer 2015; Janer 2022). Indeed, the composition of environmental research and activism on the islands reflects this discursive territorialism reproduced in academic spaces and social movements with a disproportionately marginal presence of othered subjectivities. This reflects the tensions inherent in an accelerated process of modernization and globalization that saw the population of the islands doubling through successive waves of migration, first from the Spanish mainland – this is our biographical background as *forasters –* and then foreign, partly from affluent countries, especially the UK and Germany – the *estrangers* or *guiris –* and from the Global South – primarily Latin America, Africa, and Eastern Europe – called *immigrants*. Amidst those tensions, environmental conflicts are an epitome of the contradictions of a plural and democratic society facing touristic commodification and exploitation of the natural (and social) environment.

Although there are no studies in this regard – or perhaps exactly because of that absence – we suspect that our biographies are unusual, in that we have been able to access and participate as othered peers in activist and academic spaces, despite existing entry barriers, including language, lack of social capital, and disembeddedness from nature in our early socialization from growing up in *barrios* alienated from the sea and the countryside. Ultimately, environmental movements are deliberative spaces that function as green public spheres (Torgerson 2006): reflexive, porous, and formally inclusive. However, subtle, micropolitical, capillary barriers also operate in them. The category of environmentality can capture these forms of micropower if it is thought of not just as a disciplinary governance but as a normalized regime of enunciation that prescribes who can care about the environment. Despite the apparent binarism of the categories native-foreigner and their respective association with notions of *territori* and *barrio*, it is worth reflecting on the grayness that inevitably accompanies any such dichotomy. While we still consider our experiences to be representative, they are not, as already mentioned, comprehensive. Indeed, some of the most fierce urbanization initiatives in the islands have been led by entrepreneurs with Majorcan roots (Janer 2022) while, some of the most important initiatives to conserve green spaces at the outskirts of Palma were born of neighbor associations of *barrios* from the periphery.^3^ However, the fact that such initiatives were present in the *barrio* does not mean that they were available to everyone who inhabits it.

The underlying dynamics whereby certain micropowers can regulate access through hard-to-see barriers make it difficult to pin down the specific identity dynamics at play. In the particular temporality and spatiality of our experiences, identity becomes dislocated, impure. To us, this *in-between* subject position, as Gloria Anzaldúa (2002) proposes, can have a transformative effect: the very enunciation of our voice reveals the asymmetry of access to social participation and, simultaneously, our liminality allows us to draw bridges between forms of belonging. Our own duoethnography could in a way be what Anzaldúa (2002, 557) calls an “*autohistoria”* with the germinative power of an ethos of solidarity and new legitimate claims. As Jose (Cañada 2022) explains in a reflective piece about conducting fieldwork while holding that dislocated identity, involvement in marine conservation fieldwork in the Islands helped them to start “reclaiming Catalan as [their] language, and [their] Majorcan identity as a native,” allowing a reinterpretation of the self as a legitimate user and caregiver of natural values and spaces historically “reserved for the rich and wealthy.” Joaquín’s participation in the duoethnography helped him to recognize that the inclusion of new socio-cultural profiles (such as his) has served to broaden the environmentalist agenda, bringing to the fore issues such as gentrification and challenging foundational notions of green activism and *mallorquinitat*. Still, this redrawing of intersectional divides in terms of class, language, and ethnicity holds its own limitations, as it leaves unexplored axes such as gender, age, or ability. Moreover, our voice looks and feels privileged compared to that of the migrants from the Global South, often employed in the most invisible and precarious segments of the tourist value chain (Amrhein and Langer 2025). Despite these limitations, our experiences serve as a long-exposure photograph of the articulation of *mallorquinitat* and environmental debates in the islands.

## 5 Conclusion

In this article, duoethnography emerges as a self-constitutive tool: by means of such method, we do not only redefine our own identity in relation to our field but also reconfigure and validate our ability to participate and produce change both as researchers and as citizens. In this way, a precarious method is not just a tool to produce knowledge – as methods are expected to do – but also a way to problematize constitutive hierarchies in order to intervene in reality. This form of *rebusque* (make-do) shapes inventive strategies that give us access to that which did not seem accessible from the *barrio*. Researching dynamics of privilege and marginalization in relation to environmental identity in this way connects with demands for methodological creativity and inventiveness in Latin American STS as a way to overcome academic precarity (Botero and Pérez-Bustos, this issue). Through duoethnography, we performatively and onto-epistemically challenge established discourses, practices, and barriers. Engaging with the environment is not just a way to know it, but also to shape it. Our *barrio-*based narratives of environment clash with traditional Majorcan environmentalist imaginary because they perform a different version of human–environment relations. Method here becomes a way to perform our identity in the margins by expanding the panoply of valid spaces, languages, and identities from where to care for Balearic environments. Our objective is not to substitute the dominant regional, sociolinguistic, and class character of Balearic environmentalist movements but to add diversity, offering a chance to reconfigure the debate in a way that can be more inclusive and multiple. To achieve that, visibilization of othered experiences is key, no matter how niche.

## Footnotes

1 We use the word *barrio* to denote the low- and working-class character often associated to the Spanish word, which is not necessarily present in the English word “neighbourhood.”

2 While we have discussed the Balearics as a study case, both of our histories are situated in the island of Mallorca and so we focus from now on that specific subcase.

3 For example, one of the longest initiatives for the protection of green areas is the intermittently successful struggle led by the Son Rapinya Neighbor Association since the 1980s to preserve the area of Son Quint (Parc Natural de Ponent 2020).
